# Characterization of Mucosa-Associated Microbiota in Matched Cancer and Non-neoplastic Mucosa From Patients With Colorectal Cancer

**DOI:** 10.3389/fmicb.2019.01317

**Published:** 2019-06-12

**Authors:** Polly H. M. Leung, Rao Subramanya, Qianqian Mou, Katherine Ting-wei Lee, Farhadul Islam, Vinod Gopalan, Cu-tai Lu, Alfred King-yin Lam

**Affiliations:** ^1^Department of Health Technology and Informatics, The Hong Kong Polytechnic University, Hong Kong, China; ^2^Pathology, School of Medicine, Gold Coast Campus, Griffith University, Gold Coast, QLD, Australia; ^3^Department of Biochemistry and Molecular Biology, University of Rajshahi, Rajshahi, Bangladesh; ^4^Department of Surgery, Gold Coast University Hospital, Gold Coast, QLD, Australia

**Keywords:** colorectal cancer, mucosa-associated microbiota, dysbiosis, *Fusobacterium*, *Brevundimonas*, 16S rRNA amplicon sequencing

## Abstract

Colonic microbiota play important roles in the development of colorectal cancer. We aim to characterise the mucosa-associated microbiota in the tumour as well as the matched non-neoplastic mucosa from patients with colorectal cancer. Microbiota profiling in these samples was done using high-throughput 16S rRNA amplicon sequencing. Our results showed that the microbiota richness and diversity were similar between the tumour and non-neoplastic mucosae. Linear discriminant analysis effect size (LEfSe) analysis identified *Fusobacterium* and *Campylobacter* as the key genera of the tumour while *Brevundimonas* as the key genus of the non-neoplastic mucosa. In patients with shorter survival period, the relative abundance of *Fusobacterium* and *Campylobacter* was significantly higher in the tumour. Besides, regardless of the sites, tumour showed higher abundance of *Fusobacterium*. On the other hand, the relative abundance of *Brevundimonas* was significantly lower in the tumour. When validated with quantitative ddPCR, we found the absolute numbers of both *Fusobacterium* and *F. nucleatum* were significantly higher in the carcinoma from patients with shorter survival period, conventional type of adenocarcinoma in the distal portion of the large intestine (descending colon, sigmoidal colon, and rectum). In conclusion, our study showed a compositional alteration in the mucosa-associated microbiota in the tumour, which may contribute to the progression of colorectal cancer.

## Introduction

Colorectal cancer (CRC) is the third leading cause of cancer death and accounts for 774,000 deaths worldwide in 2015 (WHO, 2018). The majority of CRC are sporadic (Amersi et al., 2005), which is also the focus for most of the research efforts. Factors associated with development of CRC involve genetic alterations, such as activation of oncogenes (Fearon and Vogelstein, 1990), loss of expression of tumour suppressor genes (Vogelstein and Kinzler, 1993), microsatellite instability and CpG methylation (Chang et al., 2002; Arends, 2013; Sakai et al., 2014). Environmental factors are correlated with the development of CRC include high fat diet, low dietary fibre, and alcohol consumption (Willett et al., 1990). As diet is closely associated with the composition of the microorganisms in the intestine, the roles of gut microbiota in the development of CRC are studied widely in the recent years (Antonic et al., 2013).

The human gastrointestinal tract is colonised with approximately 10^14^ bacteria, which comprises over 1,000 species with most of the microorganisms present in the colon (Proctor, 2011). The gut microbiota prevents colonisation of pathogenic bacteria in the intestine. They are involved in the development of the intestinal mucosal and also the wider immune system, and have an important role in the homeostasis of the intestine (Proctor, 2011). Over the last decade, with the advancement in next-generation sequencing technology, a large body of data showed that an imbalance in the microbial community occurred in colorectal adenomas and adenocarcinoma (Shen et al., 2010; Sanapareddy et al., 2012; Brim et al., 2013). Shen et al. (2010) reported a higher proportion of phylum *Proteobacteria* and lower abundance of *Bacteroidetes* in CRCs (Shen et al., 2010). Similar findings were also reported by Sanapareddy et al. (2012) that phylum *Proteobacteria*, such as *Pseudomonas*, *Helicobacter*, and *Acinetobacter*, are more abundant in colorectal adenomas (dysplasia) compared to the controls (Sanapareddy et al., 2012). These results show that alterations in the compositions of intestinal microbiota are correlated with the pathogenesis of CRC. Although the mechanisms are not well-understood, it is believed that microbiota contribute to CRC by inducing DNA damage and promoting chronic inflammation (Dulal and Keku, 2014).

So far, most of the reported studies on microbiota profiling in CRC were based on faecal samples (Wang et al., 2012; Brim et al., 2013; Wu et al., 2013; Goedert et al., 2015), however, the microbiota from faeces represent those from the intestinal lumen. Mucosa-associated microbiota are close to the intestinal epithelium and will interact directly with the colonic epithelial cells. To demonstrate if a particular microbiota composition was associated with the tumour microenvironment, we compared the microbiota in the tumour with those in the paired non-neoplastic mucosae in CRC patients using a high-throughput 16S rRNA amplicon sequencing and validated the key bacterial taxa identified using quantitative droplet digital PCR.

## Materials and Methods

### Samples

The patients recruited for this study had resection for primary colorectal adenocarcinomas between January 2012 and December 2014 in Queensland, Australia. The patients were sequentially chosen and with no selection bias. The tissues were prospectively collected fresh and rapid frozen in liquid nitrogen. In each patient, the surgeon collected one block from the tumour and one block from non-neoplastic mucosa from the proximal resection margin (as matched control) at the time of surgery. Ethics approval of this study was obtained from the Office of Human Research Ethics Committee in Gold Coast University Hospital (HREC 11 QGC 152). Nineteen pairs of matched cancer and non-cancer colonic mucosae were collected for 16S rRNA amplicon sequencing.

In each patient, the rest of the surgical section was processed in standard pathology protocol. Histological sections were cut from these tissues and stained with haematoxylin and eosin for light microscopic examination. A pathologist reported the histological parameters and pathological staging of the cancer. Every patient was managed using a standard protocol, discussed in the multidisciplinary team meetings as well as follow-up by the same team of clinicians. The clinical data and follow-up information were recorded. Table 1 summarised the features of these patients.

**TABLE 1:** Clinical and demographic details of the patients.

	**Male *n* = 9**	**Female *n* = 10**	**Total**
Age	68 ± 10.9	61.8 ± 18.6	64.7 ± 15.4
**Type of adenocarcinoma**			
Conventional adenocarcinoma	6	9	15
Mucinous adenocarcinoma	3	1	4
**Survival period**			
Below 20 months	3	4	7
20–40 months	2	4	6
Over 40 months	4	2	6
**Depth of involvement**			
T1	0	1	1
T2	0	1	1
T3	4	4	8
T4	5	4	9
**Presence of polyps in colon**			
Yes	8	5	13
No	1	5	6
**Site of tumor**			
Caecum and ascending colon, transverse colon	3	4	7
Descending colon, sigmoid colon and rectum	6	6	12

### DNA Purification

Eight 10-micron slices were sectioned from the selected frozen tissue from each case for DNA extraction. DNA samples were extracted and purified using the QIAgen DNA extraction kit (QIAgen, Hilden, Germany) according to the manufacturer’s recommendations. DNA concentrations were quantified at 260 nm using a Nanodrop Spectrophotometer (BioLab, Scoresby, VIC, Australia). DNA purity was assessed by the ratio of absorbance at 260 and 280 nm.

### PCR Amplification of V3 to V4 Regions of 16S rDNA

For identification of bacterial communities, primers specific to the variable region V3 to V4 of the 16S rDNA of bacterial species were used, the forward primer was 341F and the reverse primer was 806R. Besides, the primers also contained Illumina 5′ sequencing adapters, so that the resultant V3-V4 amplicons were incorporated with Illumina 5′ sequencing adapters. Details of the primers are shown in Supplementary Table S1. Amplification was performed using 10 μl 2× Phusion High-Fidelity PCR Master Mix (New England Biolabs, Beverly, MA, United States), 0.4 μM each primer, and 5 μl of genomic DNA. PCR conditions involved a denaturation at 98°C for 30 s, 30 cycles of denaturation at 98°C for 15 s, annealing at 60°C for 15 s, elongation at 72°C for 15 s, and final extension at 72°C for 10 min.

Suitability of the V3 and V4 primers was evaluated *in silico* using the online tool arb-SILVA TestPrime (Quast et al., 2012) based on the 16S small subunit (ssur123) and non-redundant SILVA reference database (SILVA Ref NR). At one-mismatch-stringency, the V3-V4 specific primer pairs (excluding the Illumina 5′ sequencing adapters) showed 87% coverage for all bacterial phyla and considered suitable.

### Library Preparation and Sequencing

The V3-V4 PCR products generated were 1/20-diluted, 1 μl of the diluted amplicon was used in the second PCR step. The second PCR incorporated Illumina flow-cell linkers and 8-bp dual-index barcodes to the amplicons. This was done using primers with Illumina flow cell linkers + 8-bp index barcodes + Illumina 5′ sequencing adapters. Primer sequences are shown in Supplementary Table S1. PCR was performed using 10 μl 2× Phusion High-Fidelity PCR Master Mix (New England Biolabs), 0.4 μM of each primer and 1 μl of the diluted amplicon. PCR conditions involved an initial denaturation at 98°C for 30 s, 10 cycles consisting of denaturation at 98°C for 15 s, annealing at 60°C for 15 s, elongation at 72°C for 15 s, and final extension at 72°C for 10 min.

After PCR amplification, amplicons were pooled in equal volume, the amplicon library was purified using QIAgen PCR Purification kit (QIAgen, Hilden, Germany). Quality of the library was assessed on Agilent Bioanalyser 2100 system (Agilent Technologies Inc., Santa Clara, CA, United States). The library was sequenced on an Illumina HiSeq2500 system (Illumina, San Diego, CA, United States) and 250 bp paired-end reads were generated.

### Data Processing and Bioinformatics Analysis

After sequencing, all sequence reads were processed using QIIME software (Caporaso et al., 2010). Sequences with low-quality scores, that did not perfectly match with the PCR primers and sizes were filtered. The rest of the sequences with good quality were trimmed to remove the barcode sequences, rare OTUs were also trimmed. Sequences were assembled using the FLASH software (Magoč and Salzberg, 2011) and aligned in accordance with the UPARSE software (Edgar, 2013) for assignment of operational taxonomic units (OTUs, defined at 97% sequence similarity).

The QIIME software was used to generate information on the alpha diversity including the average observed species, community richness (Chao1 index), community diversity (Shannon and Simpson indices) and sequencing depth (Good’s coverage) of each sample. The same software was also used to analyse the beta diversity (similarity) in microbial communities between the tumour and the non-neoplastic mucosa. Similarity was analysed using principal coordinate analysis (PCoA) and unweighted pair group method with arithmetic mean (UPGMA) based on weighted UniFrac distances. Analysis of similarity (ANOSIM) was used to check differences in microbiome compositions between the tumor and non-neoplastic mucosae.

The linear discriminant analysis effect size (LEfSe) was used to discover the key microbial taxa associated with the tumour and non-neoplastic mucosa. A *p-*value of < 0.05 was considered statistical significant, and the threshold linear discriminant analysis (LDA) score of > 2 was considered a discriminatory feature. An online Galaxy tool Linear Discriminant Analysis Effect Size^1^ was used for data analysis and graphical report generation.

### Droplet Digital PCR Quantification of Bacterial Genus or Species

In order to quantify the major representative bacterial taxa identified from the 16S rRNA amplicon sequencing, droplet digital PCR (ddPCR) was performed for the 19 pairs and an additional 32 pairs of matched cancer and non-cancer colonic mucosae. The clinical and demographic details of the patients are summarised in Supplementary Table S2. Primers and probes for quantification of bacterial taxa *Fusobacterium* genus, *F. nucleatum*, *Campylobacter* genus and *Brevundimonas diminuta* are shown in Supplementary Table S3. The ddPCR reaction was performed with 10 ng of DNA, 1× ddPCR Supermix for Probes (BioRad, Hercules, CA, United States), 0.25 μM of each primer, and 0.125 μM of probe in a total volume of 20 μl followed by droplet generation using a droplet generator (BioRad). The sequences of primers and probes are summarised in Supplementary Table S3. Cycling conditions included a preheating step at 95°C for 10 min followed by 45 cycles of denaturation at 94°C for 30 s, annealing for 60 s at the specific temperature for each primer pair, and final heating at 98°C for 10 min. After amplification, the PCR plate was transferred to a QX100 droplet reader (BioRad) for detection of fluorescence signals. The amplitude data were obtained by QuantaSoft software (BioRad). Bacterial strains *F. nucleatum* ATCC 25586, *Campylobacter jejuni* ATCC33560 and a laboratory strain of *Brevundimonas diminuta* previously isolated from tap water were used as positive controls for the respective primers and probes. These strains also served as negative controls mutually for the other primer pairs used in this study.

### Statistical Analyses

Wilcoxon signed rank test, Mann-Whitney *U* test and Kruskal-Wallis test were performed using Statistical Package for the Social Science (SPSS) software version 24.0 (IBM Corporation, NY, United States), where appropriate. An event was considered to be statistically significant when a *p*-value is < 0.05.

## Results

### Overview of Sequencing Data From the Tumours and Non-neoplastic Mucosae

**FIGURE 1:**
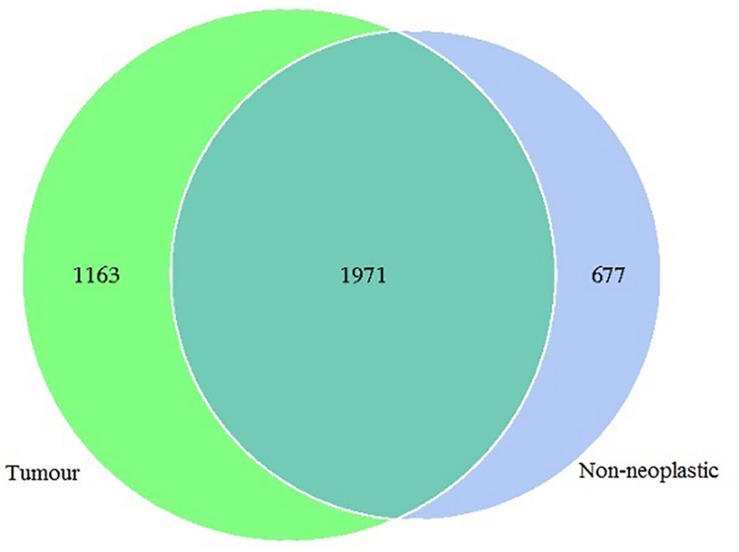
Venn diagram showing the distribution of operational taxonomic units (OTUs) detected in the tumours and non-neoplastic mucosae.

In this study, 1,565,742 tags were obtained from the 19 pairs of tissue samples with 1,498,720 (95%) of the tags annotated. The number of OTUs obtained was 3,811, with 3,134 OTUs in the tumour, 2,648 OTUs in the adjacent normal tissues, 1,971 OTUs were shared between both tumour and non-neoplastic mucosa groups (Figure 1).

### Bacterial Richness and Diversity in the Tissue Samples

To estimate the richness and diversity of bacterial communities, the alpha diversity indices were analysed. The observed species, Chao1, Shannon and Simpson’s indices were compared between the tumour and the non-neoplastic mucosa groups. Our results showed that the average observed species in the tumour and the non-neoplastic mucosal tissues were 590 ± 160 and 604 ± 124, respectively (*p* = 0.761). Chao1 indices for the tumour (754 ± 162) and non-neoplastic mucosa tissues (728 ± 213) did not differ significantly from each other (*p* = 0.511). The Shannon indices for the tumour and the non-neoplastic mucosal tissues were 5.92 ± 0.89 and 6.35 ± 0.74, respectively (*p* = 0.175). The indices for the tumour and non-neoplastic mucosa groups also did not differ significantly. The Simpson indices for the tumour and non-neoplastic mucosa tissues were 0.94 ± 0.06 and 0.96 ± 0.02, respectively (*p* = 0.148). In the pairwise comparison, there was also no significant pairwise difference in the alpha indices (*p-*values ranged from 0.316 to 0.629).

### Difference in Bacterial Compositions 0Between the Tumours and Non-neoplastic Mucosae

**FIGURE 2:**
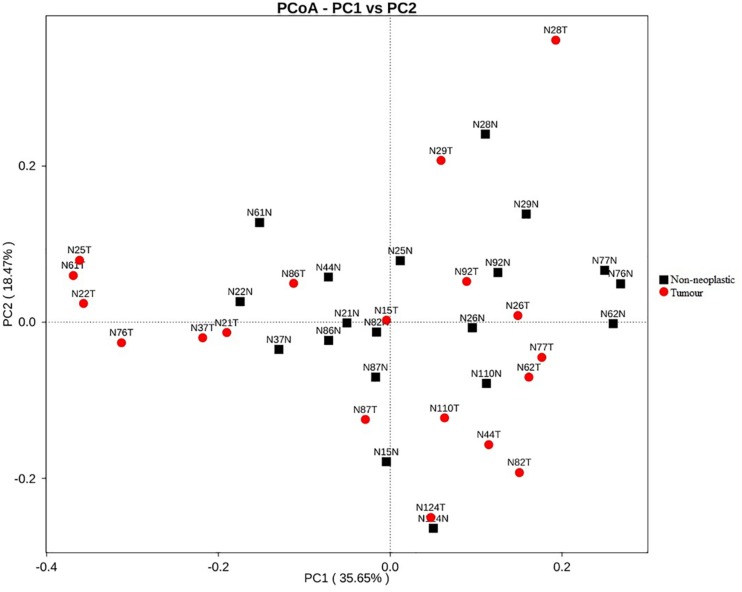
PCoA plots for weighted uniFrac distances of the variation in microbiota composition detected in the tumours and non-neoplastic mucosae.

**FIGURE 3:**
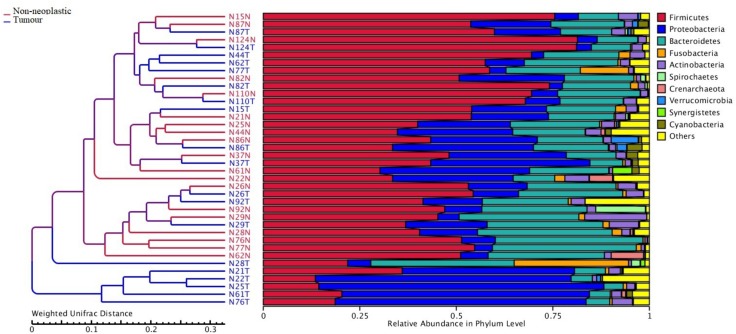
Unweighted pair group method with arithmetic mean (UPGMA) based on weighted UniFrac distances.

Apart from assessing the alpha indices, beta indices including the weighted uniFrac principal coordinate analysis (PCoA) was used to compare the similarity of the bacterial compositions in the tumour and non-neoplastic mucosal tissues. The PCoA result showed that plots of the weighted UniFrac distances did not clearly differentiate between tumour and non-neoplastic mucosa groups (Figure 2). ANOSIM showed that there was no significant difference in bacterial community in the tumor and the non-neoplastic mucosae (*p* = 0.282). The UPGMA clustering tree showed that the tumour mucosae clustered with the non-neoplastic mucosae in seven out 19 pairs of samples (N87, N124, N110, N86, N37 N26, and N29). This suggested the similarity in microbial structures between the tumour and neoplastic mucosae in these 7 pairs of samples. The same was not observed in the remaining 12 pairs of samples suggesting that the microbial structures were distinctive between tumour and non-neoplastic tissues in these samples. Notably, five tumour tissue samples (N21T, N22T, N2T, N61T, and N76T) clustered together (Figure 3), which showed the similarity in the microbial structures of the five tumour tissues. The most striking difference in this group was the increase in the relative proportion of *Proteobacteria*. When examining the clinical conditions that were common for these five patients, it was found that four out of five patients had CRC at the deepest involvement (T4).

### Bacterial Composition at the Phylum and Genus Levels

The sequences were aligned in accordance with the UPARSE software and identified to various taxonomic levels. The mean number of phylum identified from the tumour and the non-neoplastic mucosal groups were 15 and 17, respectively (*p* = 0.270). The mean number of genus identified from the tumour and the non-neoplastic mucosal groups were 119 and 123, respectively (*p* = 0.475). Although there were slightly lower numbers of phylum and genus identified in the tumour group, the differences have not reached any statistical significance.

**FIGURE 4:**
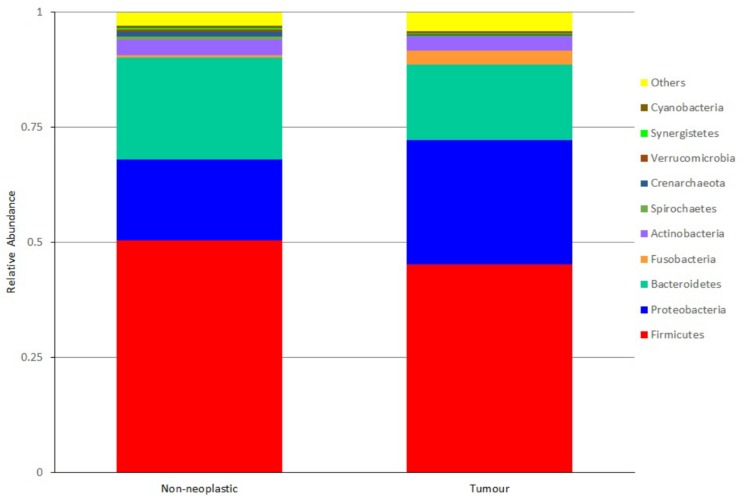
Microbiota structures in the non-neoplastic mucosae and tumours at phylum level.

At the phylum level, the most abundant phyla in both tumour and non-neoplastic mucosal tissues were *Firmicutes*, *Proteobacteria*, *Bacteroidetes*, *Fusobacteria*, and *Actinobacteria* (Figure 4). When comparing between the tumour and non-neoplastic mucosal groups, *Fusobacteria* was more abundant in the tumour (3.03% vs. 0.59% in tumours and non-neoplastic mucosae, respectively, *p* = 0.018). In pairwise comparison, *Fusobacteria* was significantly higher in the tumour (3.13% vs. 0.58% in the tumours and non-neoplastic mucosae, respectively, *p* = 0.001).

**FIGURE 5:**
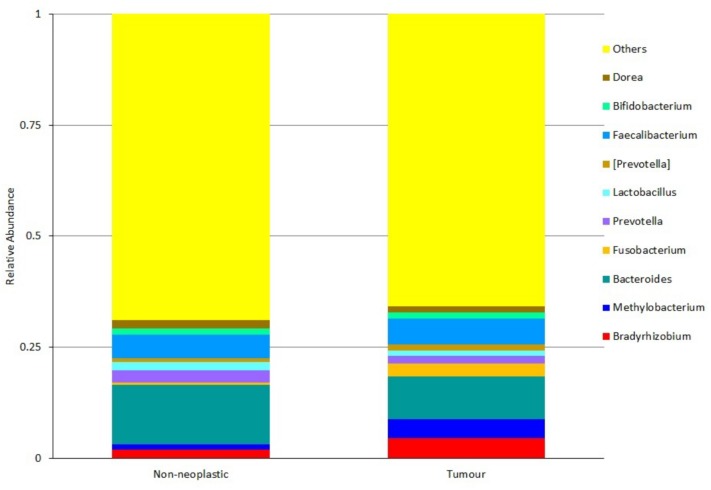
Microbiota structures in the non-neoplastic mucosae and tumours at genus level.

At the genus level, the five most predominant genera in both tumours and non-neoplastic mucosae were *Bradyrhizobium*, *Methylobacterium*, *Bacteroides*, *Fusobacterium*, and *Prevotella* (Figure 5). In the tumours, the distribution were *Bacteroides* (9.65%), *Faecalibacterium* (5.97%), *Bradyrhizobium* (4.48%), *Methylobacterium* (4.29%), and *Fusobacterium* (2.99%). In the non-neoplastic mucosae, the distribution were *Bacteroides* (13.35%), *Faecalibacterium* (5.29%), *Prevotella* (2.85%), *Bradyrhizobium* (1.96%), and *Dorea* (1.93%). Among these predominant genera, *Fusobacterium* was significantly higher in the tumours (*p* = 0.002).

For genera that were present at lower abundance, *Streptococcus* was significantly higher in the tumours than in the non-neoplastic mucosae (1.1% vs. 0.47%, *p* = 0.036). *Brevundimonas* and *Parabacteroides* were significantly lower in the tumours than in the non-neoplastic mucosae (*Brevundimonas*: 0.16% vs. 1.61%, *p* = 0.004; *Parabacteroides*: 1.41% vs. 2.19%, *p* = 0.13).

### Identification of Key Taxa Associated With Tumours and Non-neoplastic Mucosae

**FIGURE 6:**
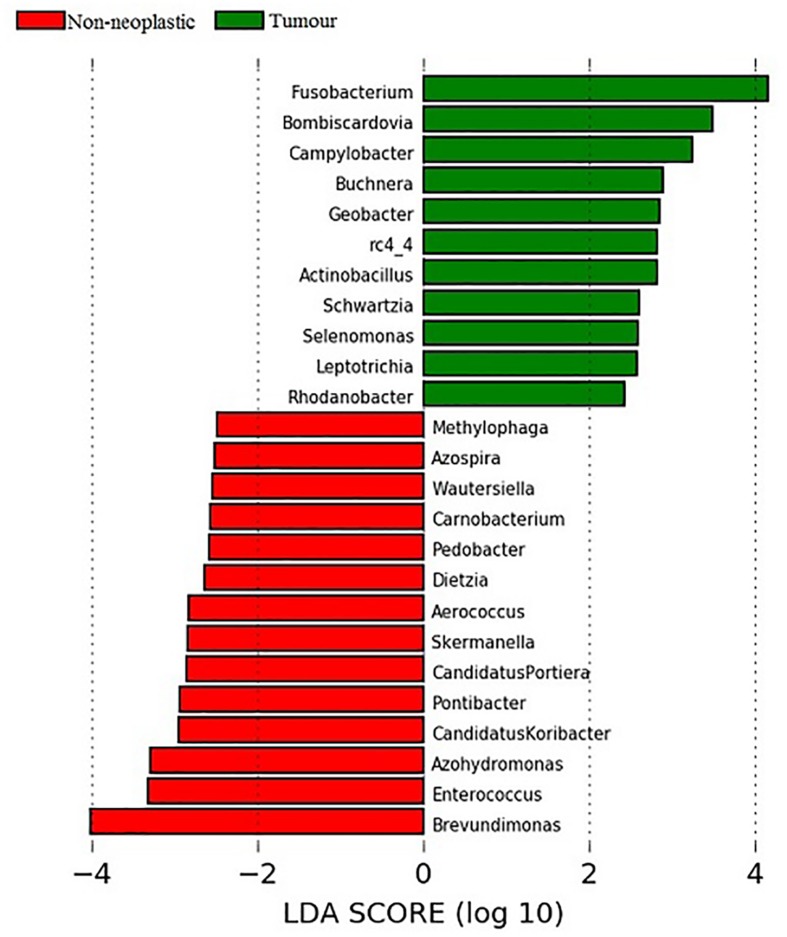
Analysis of key genera that contribute to the structure of mucosal microbiota in the non-neoplastic mucosae and tumours.

Linear discriminant analysis (LDA) coupled with effect size measurements (LEfSe) was applied to determine the key microbial taxa that were differentially represented in the tumour and normal tissues. Eleven and 14 key genera were identified in the tumours and non-neoplastic mucosae, respectively (Figure 6). Genera with mean abundance of less than 0.01% were excluded from further analysis as the read counts were low and unreliable. The key taxa identified in the tumour tissues were *Fusobacterium* (LDA score 4.15, *p* = 0.02) and *Campylobacter* (LDA score 3.48, *p* = 0.004). The key taxa in the non-neoplastic mucosae were *Brevundimonas* (LDA score 4.00, *p* = 0.01).

### Relative Abundance of the Key Taxa Under Various Clinical Conditions

To understand the relationship between the relative abundance of the key genera and characteristics of the colorectal cancer, we analysed the relative abundance of the four key genera in the tumour tissues from patients with various clinical and pathological parameters, namely survival period, carcinoma subtype (conventional vs. mucinous), presence of large intestinal polyps and location of the carcinoma. However, there was no statistical difference between the relative abundance of the key genera and these parameters.

We then performed pairwise comparison (tumour vs. non-neoplastic mucosa) in the relative abundance of the key genera from patients with the prognosis of the patients with colorectal cancer. For patients with survival time below 20 months, the relative abundance of *Fusobacterium* and *Campylobacter* was significantly higher in the tumours than the adjacent non-neoplastic mucosae (*Fusobacterium*: 2.76% vs. 0.22%, *p* = 0.018; *Campylobacter*: 0.18% vs. 0.02%, *p* = 0.018). On the other hand, *Brevundimonas* was less abundant in the tumours than the adjacent non-neoplastic mucosae (0.24% vs. 2.99%, *p* = 0.046). For patients with survival period of 20–40 months and over 40 months, there was no significant difference in the relative abundance of both genera in the tumours and non-neoplastic mucosae.

For the sub-type of adenocarcinoma, patients with conventional adenocarcinoma had significantly higher abundance of *Fusobacterium* and *Campylobacter* in the tumours than the non-neoplastic mucosae (*Fusobacterium*: 1.63% vs. 0.51%, *p* = 0.011; *Campylobacter*: 0.39% vs. 0.03%, *p* = 0.011). On the other hand, *Brevundimonas* had significantly lower abundance in the tumours than the non-neoplastic mucosae (0.16% vs. 1.88%, *p* = 0.05). For patients with mucinous adenocarcinoma, no significant difference in the abundance of the key taxa was detected between the tumours and non-neoplastic mucosae. Absence of statistical difference could be due to a small sample size (*n* = 4) of the mucinous adenocarcinoma group.

For patients with cancer and associated polyp(s) in the colon, *Brevundimonas* was present in lower abundance in the tumours than in non-neoplastic mucosae (1.3% vs. 0.01%, *p* = 0.046). In patients with cancer without associated polyp in the colon, *Fusobacterium* was significantly higher in the tumours than that in the non-neoplastic mucosae (2.85% vs. 0.67%, *p* = 0.028).

Regarding the depth of involvement, patients with CRC at the deepest involvement (T4) had higher abundance of *Campylobacter* and *Fusobacterium* in the tumour than in the non-neoplastic mucosae (*Fusobacterium*: 2.60% vs. 0.40%, *p* = 0.018; *Campylobacter*: 0.44% vs. 0.02%, *p* = 0.018).

For the tumour site, patients were divided into two groups, group 1: patients with tumours developed in the caecum, ascending and transverse colon; group 2: patients with tumours in the descending colon, sigmoidal colon and rectum. In both groups, *Fusobacterium* had significantly higher abundance in the tumours than in non-neoplastic mucosae (group 1: 2.83% vs. 0.57%, *p* = 0.046; group 2: 3.07% vs. 0.56%, *p* = 0.023). There was no statistical significance in the relative abundance of other key genera between the tumour and non-neoplastic mucosae.

### PCR Quantification of the Representative Bacterial Taxa

According to the LEfSe analysis, key taxa associated with the tumour mucosae were *Fusobacterium* genus and Campylobacter, and *Brevundimonas diminuta* were associated with non-neoplastic mucosae. To determine the absolute number of each of these bacterial taxa in the mucosae, quantitative ddPCR was performed on 19 pairs and an additional 32 pairs of matched cancer and non-cancer colonic mucosae. Apart from the *Fusobacterium* genus, the *F. nucleatum* was also included as it is a member of the *Fusobacterium* genus and is known to be significantly elevated in tumor tissues.

**FIGURE 7:**
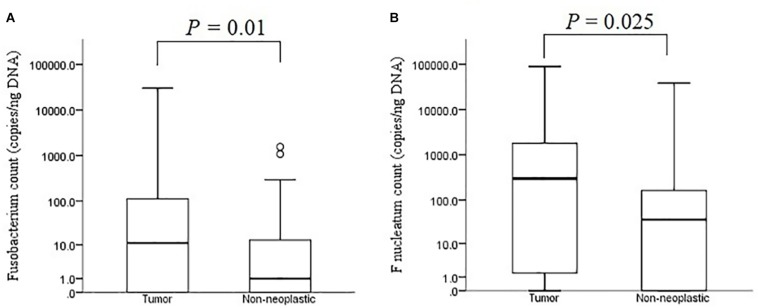
Absolute bacterial counts of *Fusobacterium* **(A)** and *F. nucleatum* **(B)** in the tumour and non-neoplastic mucosae.

The median bacterial count of *Fusobacterium* in tumour mucosae was 11 copies/ng DNA (interquartile range 0 – 124 copies/ng DNA) and that in non-neoplastic mucosae was 1 copies/ng DNA (interquartile range 0 – 13 copies/ng DNA). The difference was found to be significant (*p* = 0.01 by Mann-Whitney *U* test) (Figure 7A). For *F. nucleatum*, the median bacterial count in tumour was 298 copies/ ng DNA (interquartile range 0 – 1800 copies/ng DNA) and that in non-neoplastic mucosae was and 36 copies/ng DNA (interquartile range 0 – 172 copies/ng DNA). The difference was found to be statistically significant (*p* = 0.025 by Mann-Whitney *U* test) (Figure 7B).

*F. nucleatum* is a species within the *Fusobacterium* genus, however, we found the copy numbers of *Fusobacterium* genus were lower than those of the *F. nucleatum* species. Although ddPCR relies on end-point analysis of each droplet to generate quantitative data and amplification efficiency contributes a smaller role in the results, the use of non-specific or broad-range primers could result in lower numbers of positive droplets when compared to specific primers. The primers used for amplification of *Fusobacterium* genus were broad-range primers and it is possible that the numbers of positive droplets in the samples were lower than they should be. Nevertheless, the copy numbers of *Fusobacterium* genus in the 102 samples correlated with those of *F. nucleatum* (Spearman’s *r* = 0.830, *p* = 0.0008).

For *Campylobacter* genus, only 1 gene copy/ng DNA was detected in two tumor mucosae. For *Brevundimonas diminuta*, only 1 copy/ng DNA was detected in another two non-neoplastic mucosae. As the two bacterial taxa were present at low abundance in the tumour and non-neoplastic mucosae, taxonomic assignment would be less accurate and resulted in discrepant findings between the 16S rRNA amplicon sequencing and the quantitative ddPCR.

### Absolute Bacterial Counts and Clinical Conditions

Bacterial counts were compared pairwise between the tumour and non-neoplastic mucosae from patient under various clinical conditions (Supplementary Table S4). Significant difference in bacterial counts between tumour and non-neoplastic mucosae was observed in patients with survival period below 20 months (*Fusobacterium*: *p* = 0.01; *F. nucleatum*: *p* = 0.003). The findings were consistent with those in the 16S rRNA amplicon sequencing that *Fusobacterium* was significantly higher in the tumour mucosae of patients with survival period below 20 months.

Patients with conventional adenocarcinoma had significantly higher *Fusobacterium* and *F. nucleatum* counts in the tumours than those in the non-neoplastic mucosae (*Fusobacterium*: *p* = 0.003; *F. nucleatum*: *p* = 0.009). In contrast, patients with mucinous adenocarcinoma did not have significant difference in the bacterial counts between the tumours and non-neoplastic mucosae (*Fusobacterium*: *p* = 0.655; *F. nucleatum*: *p* = 0.593). These findings were also similar with those from the 16S rRNA amplicon sequencing. Absence of statistical difference could be due to a small sample size of the mucinous adenocarcinoma group.

For patients with associated polyps in the colon, both *Fusobacterium* and *F. nucleatum* counts in the tumours were higher than those in the non-neoplastic mucosae (*Fusobacterium*: *p* = 0.001; *F. nucleatum*: *p* = 0.004). For the depth of involvement, regardless of the stages, there was no significant difference in the number of *Fusobacterium* and *F. nucleatum* between tumour and non-neoplastic mucosae. For the tumour site, patients with tumours in the descending colon, sigmoidal colon and rectum regions had significantly higher *Fusobacterium* counts in the tumour than the neoplastic mucosae (*p* = 0.006).

## Discussion

Accumulating evidence indicates that dysbiosis and imbalance in the structure of intestinal microbiota is associated with the development of CRC (Jahani-Sherafat et al., 2018). Many studies on the relationship between intestinal microbiota and CRC were performed using faecal samples due to the non-invasiveness of sampling procedures for faecal samples. However, increase in microbial diversity was observed in the mucosa from patients with CRC (Mira-Pascual et al., 2015; Lu et al., 2016). This phenomenon was not observed in the faecal samples (Goedert et al., 2015; Mira-Pascual et al., 2015). These studies indicated that imbalance in microbiota structure was confined to the tumour tissues instead of the faecal samples. As microbiota colonising the mucosal tissues are close to the intestinal epithelium, these microorganisms are more likely to interact directly with the epithelial cells, which causes alterations in gene expression and inflammation. This may in turn influence the development of cancer (Chen et al., 2012).

In this study, we compared the microbiota composition between the tumours and the matched non-neoplastic mucosae of the large intestine. We also quantified the absolute counts of the key bacterial taxa in a bigger sample group using ddPCR. In the OTU analysis, both the tumours and non-neoplastic mucosae shared many OTUs. The alpha indices showed that the richness and diversity did not differ significantly between both tissue types. Our findings were similar to those reported in two studies with comparable study designs (Chen et al., 2012; Gao et al., 2015). In this study, the UPGMA hierarchical clustering showed that the microbiota structures were distinctive between the tumour and non-neoplastic mucosae in most of the patients. Five tumour mucosae were highly correlated and had high abundance of *Proteobacteria*. The phylum *Proteobacteria* belongs to facultative anaerobic Gram-negative bacteria, some of the genera are associated with human infections (Fierer et al., 2007). Compared to *Firmicutes* and *Bacteroides*, *Proteobacteria* is less stable and increase in abundance may be associated with inflammatory bowel diseases and CRC (Morgan et al., 2012; Wang et al., 2012). *Proteobacteria* was found to be more prevalent in patients having CRC of advanced cancer stages (Bonnet et al., 2014; Kinross et al., 2017). Within the *Proteobacteria* phylum, one of its members *Escherichia coli*, was also well-documented to be associated with CRC at stages III/IV (Bonnet et al., 2014; Kinross et al., 2017). The five cases in this study that were found to have high abundance in *Proteobacteria* belonged to CRC T4 stage.

The predominant phyla detected from both tumour and normal tissues belonged to the core microbiota, and there was overlap in the predominant genera detected from them. However, in the pairwise comparison of microbiota between the tumour and the adjacent normal tissues, the *Fusobacteria* phylum and *Fusobacterium* genus increased significantly in the tumour tissues, this genus was also identified to be a key genus of the tumour tissues in the LEfSe analysis. This was also supported by the ddPCR quantification that the absolute counts of *Fusobacterium* and *F. nucleatum* were significantly higher in the tumour than the non-noeplastic mucosae.

*Fusobacterium* is an anaerobic Gram-negative bacterium and part of the oral and intestinal flora, the organism has been found to significantly increase in CRC (Kostic et al., 2012). One of the species within the genus, *Fusobacterium nucleatum*, is highly invasive and proinflammatory, this organism is able to stimulate proliferation of cancer cells through activation of the β-catenin signalling pathway (Castellarin et al., 2012; McCoy et al., 2013). Yang et al reported that *F. nucleatum* promoted growth of CRC cells by activating nuclear factor kappa B via toll-like receptor-4 signalling in a mouse model (Yang et al., 2017).

*Campylobacter* was another key genus identified in the tumour tissues. *Campylobacter* is a Gram-negative bacterium that belongs to the *Proteobacteria* phylum. The organism is a pathogen that causes gastroenteritis in humans as well as abortion in cattle and sheep (Moore et al., 2005). The organism produces cytolethal distending toxin, a genotoxin that induces DNA breaks (Lai et al., 2016), which may interfere with the cell cycle and promote the progress of CRC. *Campylobacter* was reported to be associated with CRC as the relative abundance of the organism was found to be increased in the faecal samples from patients with CRC (Wu et al., 2013). Another study showed that the *Campylobacter* genus co-occurred with *Fusobacterium* and *Leptotrichia* in patients with CRC (Warren et al., 2013). Although *Campylobacter* was identified to be one of the key taxa in tumour mucosae using 16S rRNA amplicon sequencing, its relative abundance in the mucosal tissues was less than 1%. We were not able to detect *Campylobacter* using ddPCR. Sequences present in low abundance would affect the accuracy of taxonomic assignment in the 16S rRNA amplicon sequencing and led to disagreement with the findings from ddPCR.

In this study, we performed pairwise comparison of the abundance of key genera between the tumour and non-neoplastic mucosae in the patients with various clinicopathological status, we found *Fusobacterium* were more abundant in the tumour tissue in patients with survival period below 20 months. The same trend was observed in the patients with conventional adenocarcinomas and patients with cancer with the most extensive involvement of the large intestine (T4 stage). Our results showed that higher abundance of *Fusobacterium* in the tumour tissue was associated with advance local disease. Regardless of sites of tumour, higher abundance of *Fusobacterium* was encountered in the tumour tissues than non-neoplastic mucosae. These results also matched with the bacterial quantification results by ddPCR, except for depth of involvement, the bacterial quantification results showed no significant difference in *Fusobacterium* and *F. nucleatum* counts between tumour and non-neoplastic mucosae in T2 to T4 stages. Besides, for patients with tumours in the descending colon, sigmoidal colon and rectum regions, bacterial quantification results showed significantly higher numbers of *Fusobacterium* and *F. nucleatum* in the tumour mucosae.

*Brevundimonas* was the key genera identified in significantly greater abundance from the non-neoplastic large intestinal mucosa compared to the neoplastic tissue. However, *Brevundimonas* was not detected in most of the samples by ddPCR. The possible causes for such discrepancy could be the low relative abundance of *Brevundimonas* in the mucosae that has affected the accuracy of taxonomic assignment in the 16S rRNA amplicon sequencing. The other reason could be the use of primers targeting regions other than the V3-V4 region. *Brevundimonas* is a Gram-negative bacterium belongs to the *Proteobacteria* phylum (Segers et al., 1994). The organism is widespread in the environment including various water sources (Kulakov et al., 2002; Soto-Giron et al., 2016). *Brevundimonas* rarely causes severe human diseases but only opportunistic infections (Lee et al., 2011). So far, there is one report on the reduction of *Brevundimonas* in patients with CRC (Gao et al., 2015). In this study, we also found lower abundance of *Brevundimonas* in the tumour tissue in patients with survival below 20 months and patients with conventional adenocarcinoma. Exposure to aromatic hydrocarbon is associated with CRC (Demeyer et al., 2016), *Brevundimonas* is able to breakdown and detoxify aromatic compounds (Alkanany et al., 2017). It is possible that *Brevundimonas* reduces the toxic effect of the aromatic compounds, a reduction in the proportion of this organism may promote cancer development. However, the interaction of *Brevundimonas* with CRC cells remains to be validated.

## Conclusion

In this study, we found that the abundance of *Fusobacterium* and *Campylobacter* in the tumour mucosae were higher than those in the non-neoplastic mucosae whereas the abundance of *Brevundimonas* was lower in the tumour tissue. Following ddPCR quantification of absolute bacterial counts on more samples, we found the absolute numbers of both *Fusobacterium* and *F. nucleatum* were significantly higher in the tumour mucosae from patients with poorer prognosis, conventional type of adenocarcinoma and carcinoma in the distal portion of the large intestine (descending colon, sigmoidal colon and rectum). Our study has several limitations: first, the small sample size reduced the power of the study; second, the samples were obtained from patients of older age, which might affect the microbiota profiles; third, there was a lack of longitudinal samples from each patient, it was not known if a change in the microbiota population was the cause or consequence of CRC. Nevertheless, this study has shown that the abundance of certain bacterial genera in the tumour tissues differed significantly from that in the non-neoplastic mucosae. The imbalance of microbial population may play important roles in the pathogenesis of colorectal cancer.

## Ethics Statement

This study was carried out in accordance with the recommendations of the Human Research Ethics Committee in Gold Coast University Hospital (HREC 11 QGC 152) with written informed consent from all subjects. All subjects gave written informed consent in accordance with the Declaration of Helsinki. The protocol was approved by the Human Research Ethics Committee in Gold Coast University Hospital.

## Author Contributions

PL and AL designed the research and supervised all of the experimental works and wrote the manuscript. RS, FI, KL, VG, and C-TL collected and processed the samples. RS and QM performed the experiments. PL, AL, and RS analysed the data.

## Conflict of Interest Statement

The authors declare that the research was conducted in the absence of any commercial or financial relationships that could be construed as a potential conflict of interest.

## Footnotes

1 http://huttenhower.sph.harvard.edu/galaxy/
