# Supplementary material for: Characterization of Mucosa-Associated Microbiota in Matched Cancer and Non-neoplastic Mucosa From Patients With Colorectal Cancer
Source: Front Microbiol. 2019 Jun 12;10:1317. doi: 10.3389/fmicb.2019.01317 (PMC6581718; doi:10.3389/fmicb.2019.01317)
Supplement: Supplementary file 1 [file Table_1.DOCX]

Table S1. Primers for amplification of V3-V4 region of 16S rRNA and incorporation of index barcodes.

| Primer | Sequence (5’ – 3’) |
| --- | --- |
| Amplification of V3 to V4 regions of 16S rDNA | Illumina 5’ sequencing adapters + V3-V4-specific primers ^1^ |
| Forward  Reverse | ACACTGACGACATGGTTCTACA + CCTACGGGGGGCAGCAG  TACGGTAGCAGAGACTTGGTCT + GGACTACCGGGGTATCT |
|  |  |
| Incorporation of Illumina flow-cell linkers and 8-bp dual-index barcodes | Illumina flow cell linkers + 8-bp index barcodes + Illumina 5’ sequencing adapters |
| Forward      Reverse | AATGATACGGCGACCACCGAGATCTACAC + 8-bp index barcodes + ACACTGACGACATGGTTCTACA  CAAGCAGAAGACGGCATACGAGAT + 8-bp index barcodes + TACGGTAGCAGAGACTTGGTCT |

^1^ The universal forward and reverse primers for V3-V4 region were 341F and 806R, respectively.
