# Supplementary material for: Characterization of Mucosa-Associated Microbiota in Matched Cancer and Non-neoplastic Mucosa From Patients With Colorectal Cancer
Source: Front Microbiol. 2019 Jun 12;10:1317. doi: 10.3389/fmicb.2019.01317 (PMC6581718; doi:10.3389/fmicb.2019.01317)
Supplement: Supplementary file 2 [file Table_2.DOCX]

# Table S2. Clinical and demographic details of the 51 patients included in the droplet digital PCR for quantification of major representative bacterial taxa. These include the 19 patients for 16S rRNA amplicon sequencing and an additional 32 patients.

|  | Male  n = 26 | Female  n = 25 | Total |
| --- | --- | --- | --- |
| Age | 64.9 ± 10.0 | 63.8 ± 15.4 | 64.4 ± 12.8 |
| Type of adenocarcinoma |  |  |  |
| Conventional adenocarcinoma | 23 | 24 | 47 |
| Mucinous adenocarcinoma | 3 | 1 | 4 |
| Survival period |  |  |  |
| Below 20 months | 4 | 7 | 11 |
| 20-40 months | 12 | 9 | 21 |
| Over 40 months | 10 | 9 | 19 |
| Depth of involvement |  |  |  |
| T1 | 0 | 1 | 1 |
| T2 | 5 | 1 | 6 |
| T3 | 15 | 16 | 31 |
| T4 | 6 | 7 | 13 |
| Presence of polyps in colon |  |  |  |
| Yes | 12 | 10 | 22 |
| No | 14 | 15 | 29 |
| Site of tumor |  |  |  |
| caecum and ascending colon, transverse colon | 9 | 11 | 20 |
| descending colon, sigmoid colon and rectum | 17 | 14 | 31 |
