# Supplementary material for: Characterization of Mucosa-Associated Microbiota in Matched Cancer and Non-neoplastic Mucosa From Patients With Colorectal Cancer
Source: Front Microbiol. 2019 Jun 12;10:1317. doi: 10.3389/fmicb.2019.01317 (PMC6581718; doi:10.3389/fmicb.2019.01317)
Supplement: Supplementary file 3 [file Table_3.DOCX]

Table S3. Primers and probes for quantification of the representative bacterial taxa identified from 16S rRNA amplicon sequencing.

| Primer and probe | Sequence (5' - 3') | Target | Annealing temperature (°C) | Amplicon size (bp) | Source |
| --- | --- | --- | --- | --- | --- |
| Fusobacteria genus  f109V  f315R  Fuso-prb | CGGGTGAGTAACGCGTAAAG  GCCGTGTCTCAGTCCCCT  FAM-GAGAGAGCTTTGCGTCCCATTAGCT-BHQ1 | 16S rRNA | 66 | 207 | 1 |
| Fusobacterium nucleatum  Fn-F  Fn-R  Fn-prb | AAGCGCGTCTAGGTGGTTATGT  TGTAGTTCCGCTTACCTCTCCAG  FAM-CAACGCAATACAGAGTTGAGCCCTGCATT-BHQ1 | 16S rRNA | 60 | 108 | 2 |
| Campylobacter genus  campF2  campR2  camp-prb | CACGTGCTACAATGGCATAT  GGCTTCATGCTCTCGAGTT  FAM-CAGAGAACAATCCGAACTGGGACA-BHQ1 | 16S rRNA | 58 | 108 | 3 |
| Brevundimonas diminuta  gyrB-f  gyrB-r  gyrB-prb | ATCGAGATCATGCTGCACTATGAGGG  TGTTGTTGGTGAAGCACAGCATGG  FAM-ACGTCATCGTCATTCGCGGCCAGAA-BHQ1 | *gyrB* | 60 | 161 | 4 |

1. Walter, J., Margosch, D., Hammes, W. P., and Hertel, C. (2002). Detection of Fusobacterium species in human feces using genus-specific PCR primers and denaturing gradient gel electrophoresis. *Microb. Ecol. Health Dis.* 14, 129-132.

2. Suehiro, Y., Sakai, K., Nishioka, M., Hashimoto, S., Takami, T., Higaki, S., et al. (2017). Highly sensitive stool DNA testing of *Fusobacterium nucleatum* as a marker for detection of colorectal tumours in a Japanese population. *Ann. Clin. Biochem.* 54, 86-91.

3. Lund, M., Nordentoft, S., Pedersen, K., and Madsen, M. (2004). Detection of *Campylobacter spp*. in chicken fecal samples by real-time PCR. *J. Clin. Microbiol.* 42, 5125-5132.

4. Donofrio, R. S., Bestervelt, L. L., Saha, R., and Bagley, S. T. (2010). Quantitative real-time PCR and fluorescence in situ hybridization approaches for enumerating *Brevundimonas diminuta* in drinking water. *J. Ind. Microbiol. Biotechnol.* 37, 909-918.
