# Supplementary material for: Characterization of Mucosa-Associated Microbiota in Matched Cancer and Non-neoplastic Mucosa From Patients With Colorectal Cancer
Source: Front Microbiol. 2019 Jun 12;10:1317. doi: 10.3389/fmicb.2019.01317 (PMC6581718; doi:10.3389/fmicb.2019.01317)
Supplement: Supplementary file 4 [file Table_4.DOCX]

Supplementary Table S4. Clinical details of patients and bacterial counts in the tumour and non-neoplastic mucosae.

|  | Median bacterial count (interquartile range) of *Fusobacterium* copies/ ng DNA | | | Median bacterial count (interquartile range) of *F. nucleatum* copies/ ng DNA | | |
| --- | --- | --- | --- | --- | --- | --- |
|  | Tumour | Non-neoplastic | *p* value ^#^ | Tumour | Non-neoplastic | *p* value |
| Survival period |  |  |  |  |  |  |
| Below 20 months | 11 (2-170) | 0 (0-13) | 0.01 * | 328 (35-2155) | 8 (0-248) | 0.003 * |
| 20-40 months | 7 (0-687) | 5 (0-67) | 0.176 | 298 (0-7164) | 53 (0-1771) | 0.980 |
| Over 40 months | 5 (0-40) | 0 (0-32) | 0.374 | 29 (0-1133) | 53 (3-148) | 0.554 |
| Type of adenocarcinoma |  |  |  |  |  |  |
| Conventional adenocarcinoma | 12 (0-211) | 1 (0-15) | 0.003 * | 325 (1-2337) | 37 (0-226) | 0.009 * |
| Mucinous adenocarcinoma | 0 (0-11) | 0 (0-1) | 0.655 | 5 (0-21) | 4 (0-53) | 0.593 |
| Depth of involvement |  |  |  |  |  |  |
| T1 | 38 | 32 | ND ^@^ | 1133 | 1133 | ND |
| T2 | 2 (1-666) | 1 (0-23) | 0.268 | 111 (23-24217) | 65 (27-655) | 0.277 |
| T3 | 14 (1-124) | 1 (0-13) | 0.223 | 298 (5-2517) | 38 (0-245) | 0.336 |
| T4 | 0 (0-170) | 0 (0-19) | 0.112 | 322 (0-16566) | 4 (0-160) | 0.132 |
| Presence of polyps in colon |  |  |  |  |  |  |
| Yes | 30 (0-257) | 0 (0-4) | 0.001 * | 442 (14-4058) | 30 (0-200) | 0.004 * |
| No | 10 (0-84) | 6 (0-55) | 0.717 | 110 (0-624) | 53 (3-190) | 0.687 |
| Site of tumor |  |  |  |  |  |  |
| Caecum and ascending colon, transverse colon | 13 (1-220) | 9 (0-43) | 0.184 | 391 (15-2221) | 34 (0-250) | 0.058 |
| Descending colon, sigmoid colon and rectum | 5 (0-124) | 0 (0-4) | 0.006 * | 95 (0-1800) | 36 (0-148) | 0.086 |

# *p* values were estimated using non-parametric Wilcoxon signed-rank test.

* *p* value is significant.

^@^ ND. Not done, statistic could not be done as there was only 1 case.
